# Supplementary material for: Salivary Metabolites in Breast Cancer and Fibroadenomas: Focus on Menopausal Status and BMI
Source: Metabolites. 2024 Sep 30;14(10):531. doi: 10.3390/metabo14100531 (PMC11509358; doi:10.3390/metabo14100531)
Supplement: Supplementary file 1 [file metabolites-14-00531-s001.zip › Bel'skaya_Supplementary.pdf]

Table 1S. Values of biochemical parameters of saliva in breast cancer, fibroadenomas and healthy controls depending on menopause status

Indicators	MP	Breast cancer	Fibroadenomas	Healthy controls	Kruskal-Wallis test (H, p)
Age, years	No	43.0 [37.0; 47.0]	34.0 [27.0; 42.0]	37.0 [31.0; 41.0]	88.03; 0.0000
	Yes	60.0 [55.0; 65.0]	57.5 [52.0; 63.0]	57.0 [53.0; 62.0]	16.27; 0.0003
BMI	No	25.39 [21.63; 29.52]	23.32 [20.20; 27.22]	-	0.0003
	Yes	30.07 [26.56; 33.32]	30.07 [26.11; 33.67]	-	0.9490
pH	No	6.42 [6.19; 6.64]	6.48 [6.26; 6.64]	6.43 [6.22; 6.65]	4.450; 0.1081
	Yes	6.51 [6.28; 6.76]	6.55 [6.33; 6.80]	6.57 [6.36; 6.78]	1.796; 0.4074
Ca	No	1.13 [0.73; 1.49]	1.20 [0.93; 1.58]	1.22 [0.98; 1.52]	6.160; 0.0460
	Yes	1.29 [0.94; 1.69]	1.32 [1.01; 1.70]	1.30 [1.05; 1.60]	1.667; 0.5579
P	No	4.33 [3.24; 5.63]	4.18 [3.37; 5.18]	4.08 [3.15; 5.42]	0.8417; 0.6565
	Yes	4.68 [3.54; 6.02]	4.32 [3.43; 5.42]	4.57 [3.59; 5.95]	5.001; 0.0821
Na	No	6.61 [4.57; 13.09]	7.03 [4.86; 11.54]	8.22 [5.55; 12.41]	3.513; 0.1727
	Yes	8.17 [5.08; 12.49]	6.83 [4.83; 11.00]	8.63 [5.56; 12.01]	3.419; 0.1810
K	No	10.29 [7.97; 13.59]	10.38 [8.34; 12.93]	11.08 [8.76; 13.51]	4.570; 0.1018
	Yes	11.91 [8.12; 14.92]	11.41 [8.42; 14.72]	10.59 [8.65; 14.90]	0.6792; 0.7121
Cl	No	25.05 [19.61; 32.91]	22.98 [19.26; 27.66]	23.91 [20.00; 29.88]	7.717; 0.0211
	Yes	26.21 [19.78; 31.76]	25.40 [20.71; 31.13]	26.67 [21.74; 32.50]	1.894; 0.3879
Mg	No	0.282 [0.210; 0.380]	0.276 [0.209; 0.337]	0.286 [0.232; 0.349]	4.288; 0.1172
	Yes	0.303 [0.245; 0.377]	0.288 [0.219; 0.372]	0.305 [0.244; 0.356]	1.950; 0.3772
Protein	No	0.56 [0.32; 1.01]	0.64 [0.39; 0.96]	0.98 [0.58; 1.43]	49.13; 0.0000
	Yes	0.67 [0.40; 1.05]	0.72 [0.50; 1.17]	1.20 [0.68; 2.04]	55.47; 0.0000
Urea	No	8.22 [5.70; 11.96]	7.39 [5.00; 10.37]	6.40 [4.07; 8.95]	17.35; 0.0002
	Yes	10.30 [7.07; 13.94]	9.39 [6.84; 12.68]	6.76 [4.84; 9.29]	49.79; 0.0000
UA	No	54.82 [20.64; 108.87]	74.77 [32.69; 116.82]	64.22 [25.23; 126.15]	7.395; 0.0248
	Yes	64.29 [21.93; 137.61]	77.96 [34.72; 144.44]	102.82 [48.17; 182.02]	18.27; 0.0001
Albumin	No	0.276 [0.138; 0.439]	0.256 [0.162; 0.402]	0.224 [0.147; 0.358]	2.643; 0.2668
	Yes	0.308 [0.184; 0.550]	0.351 [0.199; 0.595]	0.337 [0.212; 0.532]	1.581; 0.4537
ALT	No	4.08 [2.92; 5.54]	4.00 [2.62; 5.46]	4.00 [2.87; 5.19]	1.789; 0.4089
	Yes	4.08 [3.00; 5.46]	3.73 [2.77; 5.35]	3.77 [2.69; 4.85]	6.977; 0.0306
AST	No	6.00 [4.42; 9.00]	5.83 [4.00; 8.00]	5.33 [3.83; 7.33]	6.225; 0.0445
	Yes	6.33 [4.42; 8.50]	6.08 [4.13; 7.63]	6.00 [3.50; 7.92]	4.737; 0.0936
AST/ALT	No	1.40 [1.10; 1.88]	1.44 [1.13; 1.82]	1.32 [1.08; 1.68]	3.533; 0.1709
	Yes	1.51 [1.17; 1.95]	1.42 [1.16; 1.84]	1.50 [1.18; 2.07]	1.630; 0.4425
α-AAs	No	4.15 [3.86; 4.65]	4.11 [3.87; 4.46]	4.03 [3.81; 4.26]	11.85; 0.0027
	Yes	4.26 [3.88; 4.81]	4.23 [3.97; 4.73]	4.13 [3.87; 4.45]	7.051; 0.0294
ICs	No	0.330 [0.212; 0.448]	0.311 [0.205; 0.432]	0.334 [0.235; 0.432]	0.7383; 0.6913
	Yes	0.266 [0.167; 0.379]	0.258 [0.152; 0.364]	0.281 [0.190; 0.379]	3.344; 0.1878
NO	No	28.86 [17.72; 42.98]	33.33 [18.42; 55.79]	22.98 [12.63; 34.39]	27.10; 0.0000
	Yes	28.16 [18.33; 41.75]	32.11 [18.42; 55.79]	22.63 [13.33; 42.63]	9.591; 0.0083
ALP	No	73.88 [49.98; 108.65]	69.54 [47.81; 97.79]	58.67 [39.11; 80.40]	15.18; 0.0005
	Yes	73.88 [47.81; 106.48]	72.80 [45.63; 98.87]	63.02 [45.63; 91.27]	3.352; 0.1871
MM 254	No	0.239 [0.158; 0.325]	0.230 [0.166; 0.319]	0.263 [0.169; 0.348]	3.367; 0.1857
	Yes	0.263 [0.167; 0.402]	0.264 [0.169; 0.364]	0.287 [0.183; 0.397]	2.276; 0.2624

MM280	No	0.185 [0.128; 0.281]	0.195 [0.139; 0.270]	0.207 [0.139; 0.305]	1.411; 0.4939
	Yes	0.209 [0.142; 0.342]	0.206 [0.150; 0.330]	0.240 [0.164; 0.356]	3.435; 0.1795
LDH	No	1419.0 [767.3; 2008.0]	1390.0 [860.0; 1940.0]	1008.0 [592.8; 1732.0]	12.64; 0.0018
	Yes	1564.0 [895.9; 2090.0]	1432.0 [831.6; 2068.0]	1269.0 [684.0; 2028.0]	4.148; 0.1257
CAT	No	3.44 [2.34; 5.39]	3.50 [2.42; 5.05]	4.14 [2.24; 5.53]	3.442; 0.1789
	Yes	3.90 [2.64; 6.07]	3.98 [2.63; 6.11]	4.89 [3.88; 6.23]	14.18; 0.0008
SAs	No	0.189 [0.140; 0.269]	0.183 [0.122; 0.256]	0.183 [0.134; 0.299]	3.536; 0.1707
	Yes	0.207 [0.134; 0.275]	0.171 [0.122; 0.232]	0.183 [0.128; 0.287]	7.295; 0.0261
PYR	No	13.97 [10.29; 18.38]	12.50 [9.07; 17.89]	12.01 [9.31; 16.91]	3.263; 0.1956
	Yes	14.71 [9.93; 20.10]	13.97 [9.80; 19.12]	13.97 [9.56; 19.85]	0.5329; 0.7661
DC	No	4.02 [3.79; 4.23]	3.95 [3.75; 4.17]	3.94 [3.78; 4.03]	8.514; 0.0142
	Yes	3.93 [3.72; 4.18]	3.97 [3.75; 4.15]	3.90 [3.79; 4.10]	0.2152; 0.8980
TC	No	0.869 [0.774; 0.987]	0.901 [0.806; 1.030]	0.891 [0.817; 0.965]	4.502; 0.1053
	Yes	0.904 [0.810; 1.021]	0.887 [0.791; 0.993]	0.896 [0.829; 1.015]	3.115; 0.2106
SB	No	0.535 [0.491; 0.612]	0.556 [0.507; 0.643]	0.546 [0.510; 0.576]	8.736; 0.0127
	Yes	0.542 [0.483; 0.674]	0.543 [0.488; 0.641]	0.545 [0.510; 0.578]	0.0755; 0.9630
MDA	No	6.75 [5.64; 8.55]	7.01 [5.90; 8.89]	6.41 [5.56; 7.78]	14.85; 0.0006
	Yes	6.92 [5.47; 8.80]	7.26 [5.81; 9.15]	6.67 [5.90; 8.08]	3.301; 0.1919
GGT	No	22.6 [20.0; 25.3]	21.3 [18.7; 24.2]	19.1 [17.0; 24.1]	24.90; 0.0000
	Yes	23.7 [20.0; 26.9]	21.9 [19.6; 25.1]	21.7 [18.1; 25.2]	12.82; 0.0016
SM	No	0.094 [0.060; 0.139]	0.101 [0.070; 0.144]	0.083 [0.058; 0.122]	10.37; 0.0056
	Yes	0.097 [0.062; 0.154]	0.105 [0.062; 0.157]	0.102 [0.073; 0.138]	0.5916; 0.7439
SOD	No	68.4 [31.6; 142.1]	60.5 [31.6; 107.9]	57.9 [31.6; 123.7]	1.843; 0.3980
	Yes	73.7 [36.8; 142.1]	65.8 [36.8; 123.7]	60.5 [31.6; 105.3]	4.442; 0.1085
α-Amylase	No	344.6 [134.2; 564.8]	241.0 [110.6; 416.5]	171.4 [76.5; 331.2]	13.09; 0.0014
	Yes	304.9 [116.5; 636.0]	243.8 [122.4; 591.6]	234.9 [100.8; 502.8]	2.003; 0.3673
Lactate	No	2.14 [1.29; 2.85]	2.11 [1.50; 3.03]	2.33 [1.47; 3.40]	4.520; 0.1043
	Yes	2.42 [1.49; 3.54]	2.40 [1.58; 3.36]	2.27 [1.41; 3.97]	0.0558; 0.9725
AOA	No	1.60 [1.31; 1.95]	1.67 [1.41; 1.99]	1.80 [1.57; 2.40]	3.522; 0.1719
	Yes	1.70 [1.45; 2.14]	1.54 [1.32; 1.84]	1.77 [1.48; 2.23]	11.72; 0.0028
Peroxidase	No	0.500 [0.250; 0.920]	0.415 [0.190; 0.715]	0.245 [0.120; 0.480]	7.538; 0.0231
	Yes	0.420 [0.250; 0.830]	0.490 [0.250; 0.810]	0.540 [0.280; 0.920]	0.7821; 0.6764
MM280/254	No	0.844 [0.764; 0.950]	0.866 [0.778; 0.959]	0.827 [0.732; 0.956]	4.565; 0.1020
	Yes	0.860 [0.775; 0.962]	0.866 [0.775; 0.968]	0.853 [0.773; 0.941]	0.5087; 0.7754

Note. MP – menopause, Ca – calcium, P – phosphorus, Na – sodium, K – potassium, Cl – chlorides, Mg – magnesium, UA – uric acid, ALT – alanine aminotransferase, AST – aspartate aminotransferase, α-AAs – total α-amino acid content, ICs – Imidazole compounds, NO – nitric oxide, ALP – alkaline phosphatase, MM – medium molecular weight toxins, LDH - lactate dehydrogenase, CAT – catalase, SAs – sialic acids, PYR – pyruvic acid, DC – diene conjugates, TC – triene conjugates, SB – Schiff bases, MDA – malondialdehyde, GGT – gamma glutamyl transferase, SM – seromucoids, SOD – superoxide dismutase, AOA – antioxidant activity.

Table 2S. Statistical significance (p-value) of differences in biochemical parameter values in subgroups (BC, FA, HC) depending on menopause status

Indicators	Breast cancer (BC)	Fibroadenomas (FA)	Healthy controls (HC)
pH	0.0007	0.0053	0.0001
Ca	0.0032	0.0187	0.1939
P	0.0552	0.4088	0.0903
Na	0.3788	0.8898	0.6997
K	0.0193	0.0652	0.9048
Cl	0.4294	0.0004	0.0073
Mg	0.2299	0.0874	0.3549
Protein	0.0163	0.0053	0.0013
Urea	0.0004	0.0000	0.1705
UA	0.0671	0.1122	0.0002
Albumin	0.0486	0.0000	0.0001
ALT	0.9728	0.7499	0.2013
AST	0.9783	0.8936	0.2674
AST/ALT	0.2884	0.9351	0.0096
α-AAs	0.2404	0.0033	0.0285
ICs	0.0005	0.0001	0.0221
NO	0.8894	0.9892	0.4118
ALP	0.7246	0.8593	0.0607
MM 254	0.0562	0.0502	0.0810
MM280	0.0490	0.0510	0.0173
LDH	0.1139	0.7530	0.0405
CAT	0.0263	0.0159	0.0009
SAs	0.5735	0.2917	0.5242
PYR	0.3959	0.0584	0.0496
DC	0.0505	0.9861	0.7452
TC	0.0137	0.1538	0.7136
SB	0.2702	0.1761	0.8552
MDA	0.8784	0.7361	0.0485
GGT	0.0915	0.0102	0.0045
SM	0.2801	0.4355	0.0108
SOD	0.6038	0.2966	0.7932
α-Amylase	0.9937	0.4603	0.0079
Lactate	0.0523	0.1138	0.7829
AOA	0.0228	0.0210	0.7122
Peroxidase	0.5845	0.0780	0.0101
MM280/254	0.4020	0.8773	0.2400

Table 3S. Biochemical composition of saliva in breast cancer depending on menopause status and BMI

Indicators	No menopause			Menopause	Kruskal-Wallis test (H, p)
	BMI <25	BMI >25	p-value		
Age, years	40.0 [35.0; 45.0]	44.0 [39.5; 48.0]	0.0002	60.0 [55.0; 65.0]	293.8; 0.0000
BMI	21.50 [19.94; 23.12]	29.12 [26.47; 32.74]	0.0000	30.07 [26.56; 33.32]	172.0; 0.0000
pH	6.43 [6.17; 6.72]	6.39 [6.22; 6.59]	0.6893	6.51 [6.28; 6.76]	10.97; 0.0042
Ca	1.10 [0.72; 1.45]	1.13 [0.78; 1.57]	0.5694	1.29 [0.94; 1.69]	9.605; 0.0082
P	4.08 [3.05; 5.64]	4.38 [3.50; 5.56]	0.5918	4.68 [3.54; 6.02]	3.905; 0.1420
Na	5.50 [4.54; 11.05]	8.24 [5.18; 14.85]	0.0601	8.17 [5.08; 12.49]	4.438; 0.1087
K	9.58 [7.27; 11.47]	10.64 [8.35; 14.18]	0.0623	11.91 [8.12; 14.92]	8.819; 0.0122
Cl	22.75 [19.76; 28.33]	26.83 [19.91; 35.24]	0.0600	26.21 [19.78; 31.76]	4.562; 0.1022
Mg	0.281 [0.194; 0.379]	0.282 [0.219; 0.375]	0.7590	0.303 [0.245; 0.377]	2.067; 0.3558
Protein	0.56 [0.33; 0.98]	0.54 [0.30; 1.11]	0.9454	0.67 [0.40; 1.05]	5.752; 0.0564
Urea	7.47 [4.96; 10.51]	9.71 [5.90; 13.33]	0.0092	10.30 [7.07; 13.94]	20.94; 0.0000
UA	55.77 [25.00; 97.50]	52.30 [15.77; 132.50]	0.8179	64.29 [21.93; 137.61]	3.0494 0.2177
Albumin	0.255 [0.124; 0.388]	0.293 [0.170; 0.590]	0.1071	0.308 [0.184; 0.550]	6.307; 0.0427
ALT	4.00 [2.92; 5.38]	4.23 [2.77; 6.85]	0.4174	4.08 [3.00; 5.46]	0.5789; 0.7489
AST	5.96 [4.58; 9.42]	6.50 [4.42; 8.83]	0.9605	6.33 [4.42; 8.50]	0.0098; 0.9951
AST/ALT	1.54 [1.08; 1.87]	1.33 [1.14; 1.89]	0.5524	1.51 [1.17; 1.95]	1.173; 0.5563
α-AAs	4.11 [3.86; 4.56]	4.21 [3.91; 4.88]	0.2071	4.26 [3.88; 4.81]	2.642; 0.2668
ICs	0.341 [0.235; 0.448]	0.326 [0.205; 0.448]	0.4454	0.266 [0.167; 0.379]	13.80; 0.0010
NO	28.77 [17.02; 42.46]	26.84 [17.72; 45.61]	0.6524	28.16 [18.33; 41.75]	0.1113; 0.9458
ALP	71.71 [49.98; 99.96]	80.40 [48.89; 110.82]	0.4313	73.88 [47.81; 106.48]	0.7731; 0.6794
MM 254	0.213 [0.160; 0.315]	0.244 [0.148; 0.355]	0.7497	0.263 [0.167; 0.402]	3.297; 0.1923
MM280	0.180 [0.146; 0.277]	0.201 [0.112; 0.289]	0.8808	0.209 [0.142; 0.342]	3.274; 0.1945
LDH	1419.0 [836.0; 2007.0]	1445.5 [640.7; 2028.0]	0.7970	1564.0 [895.9; 2090.0]	2.348; 0.3091
CAT	3.70 [2.60; 5.49]	3.30 [2.17; 4.96]	0.3744	3.90 [2.64; 6.07]	5.373; 0.0681
SAs	0.195 [0.140; 0.269]	0.189 [0.140; 0.275]	0.8809	0.207 [0.134; 0.275]	0.3909; 0.8225
PYR	14.95 [10.29; 18.63]	13.73 [10.29; 17.89]	0.2549	14.71 [9.93; 20.10]	1.621; 0.4447
DC	4.04 [3.82; 4.28]	3.98 [3.78; 4.21]	0.3806	3.93 [3.72; 4.18]	5.051; 0.0800
TC	0.869 [0.759; 1.000]	0.862 [0.780; 0.971]	0.9174	0.904 [0.810; 1.021]	6.548; 0.0378
SB	0.539 [0.494; 0.629]	0.524 [0.465; 0.612]	0.2974	0.542 [0.483; 0.674]	2.376; 0.3049
MDA	6.92 [5.81; 8.80]	6.58 [5.56; 8.55]	0.2298	6.92 [5.47; 8.80]	1.212; 0.5455
GGT	21.6 [19.0; 25.0]	23.0 [20.7; 26.4]	0.0452	23.7 [20.0; 26.9]	6.673; 0.0356
SM	0.104 [0.064; 0.149]	0.086 [0.057; 0.128]	0.1677	0.097 [0.062; 0.154]	2.605; 0.2719
SOD	67.1 [39.5; 152.6]	71.1 [26.3; 139.5]	0.5272	73.7 [36.8; 142.1]	0.6256; 0.7314
α-Amylase	522.2 [177.7; 798.0]	220.8 [115.0; 450.1]	0.0331	304.9 [116.5; 636.0]	4.351; 0.1136
Lactate	1.79 [1.27; 2.81]	2.29 [1.48; 2.92]	0.3426	2.42 [1.49; 3.54]	4.606; 0.0999
AOA	1.64 [1.38; 2.10]	1.54 [1.30; 1.90]	0.2281	1.70 [1.45; 2.14]	6.286; 0.0432
Peroxidase	0.560 [0.340; 1.085]	0.430 [0.230; 0.760]	0.2356	0.420 [0.250; 0.830]	1.876; 0.3914
MM280/254	0.844 [0.768; 0.962]	0.846 [0.759; 0.948]	0.4675	0.860 [0.775; 0.962]	0.0994; 0.6067

Table 4S. Biochemical composition of saliva in fibroadenomas depending on menopause status and BMI

Indicators	No menopause			Menopause	Kruskal-Wallis test (H, p)
	BMI <25	BMI <25	p-value		
Age, years	31.0 [24.5; 38.0]	41.0 [34.0; 45.0]	0.0000	57.5 [52.0; 63.0]	361.9; 0.0000
BMI	20.70 [19.41; 22.74]	28.96 [26.40; 31.64]	0.0000	30.07 [26.11; 33.67]	397.5; 0.0000
pH	6.52 [6.29; 6.64]	6.44 [6.23; 6.64]	0.0757	6.55 [6.33; 6.80]	10.36; 0.0056
Ca	1.19 [0.91; 1.53]	1.25 [0.95; 1.70]	0.1272	1.32 [1.01; 1.70]	8.164; 0.0169
P	4.17 [3.25; 5.20]	4.17 [3.56; 5.14]	0.4856	4.32 [3.43; 5.42]	1.254; 0.5342
Na	6.42 [4.70; 10.80]	8.21 [5.16; 14.11]	0.0221	6.83 [4.83; 11.00]	5.237; 0.0729
K	10.14 [8.29; 12.52]	10.92 [8.68; 14.03]	0.1546	11.41 [8.42; 14.72]	5.278; 0.0714
Cl	22.42 [18.85; 27.29]	23.53 [19.69; 28.91]	0.0524	25.40 [20.71; 31.13]	16.64; 0.0002
Mg	0.278 [0.207; 0.338]	0.270 [0.213; 0.334]	0.8203	0.288 [0.219; 0.372]	3.155; 0.2065
Protein	0.62 [0.35; 0.87]	0.67 [0.43; 1.09]	0.0289	0.72 [0.50; 1.17]	13.29; 0.0013
Urea	6.96 [4.82; 10.23]	7.89 [5.07; 11.30]	0.1806	9.39 [6.84; 12.68]	20.93; 0.0000
UA	76.61 [31.93; 115.38]	71.15 [34.62; 119.23]	0.9310	77.96 [34.72; 144.44]	2.303; 0.3162
Albumin	0.240 [0.161; 0.357]	0.270 [0.170; 0.473]	0.0425	0.351 [0.199; 0.595]	22.20; 0.0000
ALT	3.92 [2.62; 5.38]	4.15 [2.77; 5.62]	0.2203	3.73 [2.77; 5.35]	1.601; 0.4492
AST	5.75 [3.92; 7.83]	6.04 [4.08; 8.08]	0.6286	6.08 [4.13; 7.63]	0.2695; 0.8795
AST/ALT	1.47 [1.15; 1.91]	1.38 [1.12; 1.72]	0.0852	1.42 [1.16; 1.84]	3.060; 0.2165
α-AAs	4.09 [3.86; 4.42]	4.16 [3.90; 4.54]	0.1639	4.23 [3.97; 4.73]	10.95; 0.0042
ICs	0.315 [0.212; 0.440]	0.303 [0.190; 0.455]	0.5600	0.258 [0.152; 0.364]	16.87; 0.0002
NO	34.12 [19.21; 56.93]	31.75 [18.25; 55.09]	0.3685	32.11 [18.42; 55.79]	0.7927; 0.6728
ALP	67.36 [45.63; 93.44]	73.88 [52.15; 117.34]	0.0496	72.80 [45.63; 98.87]	3.991; 0.1360
MM 254	0.215 [0.157;0.310]	0.249 [0.173; 0.352]	0.0327	0.264 [0.169; 0.364]	8.005; 0.0183
MM280	0.189 [0.137; 0.264]	0.204 [0.151; 0.288]	0.0904	0.206 [0.150; 0.330]	6.167; 0.0454
LDH	1281.5 [806.6; 1881.5]	1469.0 [876.5; 2037.0]	0.1007	1432.0 [831.6; 2068.0]	2.690; 0.2605
CAT	3.44 [2.49; 4.70]	3.70 [2.28; 5.15]	0.7869	3.98 [2.63; 6.11]	6.046; 0.0487
SAs	0.177 [0.122; 0.250]	0.195 [0.128; 0.262]	0.5296	0.171 [0.122; 0.232]	1.562; 0.4580
PYR	12.50 [8.82; 17.40]	12.99 [9.31; 18.38]	0.5390	13.97 [9.80; 19.12]	3.849; 0.1459
DC	3.94 [3.73; 4.18]	3.96 [3.81; 4.10]	0.8149	3.97 [3.75; 4.15]	0.0599; 0.9705
TC	0.893 [0.802; 1.003]	0.914 [0.812; 1.066]	0.0651	0.887 [0.791; 0.993]	5.249; 0.0725
SB	0.557 [0.507; 0.635]	0.556 [0.501; 0.664]	0.7761	0.543 [0.488; 0.641]	1.918; 0.3832
MDA	7.01 [5.90; 8.97]	7.05 [5.98; 8.55]	0.9891	7.26 [5.81; 9.15]	0.0327; 0.9838
GGT	21.2 [18.5; 24.0]	21.5 [18.9; 24.3]	0.4183	21.9 [19.6; 25.1]	6.994; 0.0303
SM	0.100 [0.069; 0.146]	0.101 [0.072; 0.144]	0.6338	0.105 [0.062; 0.157]	0.8607; 0.6503
SOD	60.5 [31.6; 102.6]	59.2 [32.9; 107.9]	0.7948	65.8 [36.8; 123.7]	1.228; 0.5411
α-Amylase	249.1 [117.9; 392.9]	207.4 [106.1; 454.2]	0.8269	243.8 [122.4; 591.6]	0.6253; 0.7315
Lactate	2.08 [1.51; 3.12]	2.12 [1.40; 2.89]	0.4040	2.40 [1.58; 3.36]	3.323; 0.1898
AOA	1.69 [1.41; 1.99]	1.62 [1.41; 2.00]	0.5680	1.54 [1.32; 1.84]	5.764; 0.0560
Peroxidase	0.440 [0.220; 0.665]	0.340 [0.170; 0.730]	0.4003	0.490 [0.250; 0.810]	3.979; 0.1367
MM280/254	0.873 [0.788; 0.972]	0.852 [0.763; 0.936]	0.1905	0.866 [0.775; 0.968]	1.674; 0.4330

Table 5S. Comparison of the biochemical composition of saliva in leaf-shaped (phyllodes) tumors and fibroadenomas before menopause

Indicators	Leaf-shaped (phyllodes) tumor, n=55 (1)	FA (no menopause), n=379 (2)	(1) vs. (2)	(1) vs. BC	(2) vs. BC
pH	6.48 [6.29; 6.60]	6.47 [6.25; 6.64]	0.8888	0.2054	0.0813
Ca	1.16 [0.92; 1.47]	1.21 [0.93; 1.58]	0.6273	0.2477	0.0215
P	4.15 [3.60; 4.85]	4.19 [3.31; 5.25]	0.8011	0.4452	0.3874
Na	8.16 [5.41; 14.84]	6.89 [4.78; 10.93]	0.0871	0.1918	0.7629
K	9.87 [7.70; 11.75]	10.53 [8.41; 13.12]	0.1292	0.4041	0.4752
Cl	22.42 [17.45; 26.40]	23.06 [19.35; 27.69]	0.2360	0.0215	0.0277
Mg	0.255 [0.217; 0.346]	0.278 [0.207; 0.336]	0.6562	0.2385	0.1612
Protein	0.55 [0.35; 0.80]	0.64 [0.40; 1.00]	0.0960	0.7699	0.0874
Urea	6.11 [4.73; 9.81]	7.70 [5.02; 10.71]	0.1885	0.0330	0.0839
UA	73.91 [38.10; 115.38]	74.77 [31.61; 116.97]	0.9381	0.1043	0.0063
Albumin	0.235 [0.152; 0.378]	0.258 [0.162; 0.404]	0.4800	0.3453	0.5420
ALT	3.92 [2.92; 6.31]	4.00 [2.62; 5.38]	0.2773	0.9765	0.1410
AST	6.67 [4.92; 8.33]	5.83 [4.00; 7.83]	0.0695	0.7557	0.0540
AST/ALT	1.45 [1.15; 2.04]	1.44 [1.13; 1.81]	0.9080	0.7890	0.9732
α-AAs	4.17 [3.88; 4.36]	4.11 [3.87; 4.48]	0.8185	0.5033	0.3201
ICs	0.303 [0.175; 0.486]	0.311 [0.205; 0.432]	0.9761	0.6208	0.4289
NO	29.30 [15.61; 55.26]	33.33 [18.60; 56.14]	0.5364	0.5478	0.0477
ALP	69.54 [47.81; 106.48]	69.54 [47.81; 97.79]	0.6261	0.7786	0.2109
MM 254	0.245 [0.152; 0.321]	0.230 [0.168; 0.319]	0.9205	0.8276	0.8632
MM280	0.193 [0.139; 0.265]	0.195 [0.139; 0.271]	0.9279	0.5871	0.6131
LDH	1155.0 [747.8; 1914.0]	1412.0 [870.4; 1979.0]	0.5421	0.7940	0.8188
CAT	3.69 [2.28; 5.15]	3.45 [2.42; 5.01]	0.7600	0.8386	0.9957
SAs	0.177 [0.122; 0.244]	0.183 [0.122; 0.256]	0.5533	0.1497	0.2078
PYR	14.83 [9.93; 19.98]	12.25 [9.07; 17.40]	0.1110	0.6602	0.0832
DC	4.10 [3.86; 4.23]	3.94 [3.74; 4.14]	0.0055	0.3525	0.0136
TC	0.892 [0.811; 0.997]	0.901 [0.802; 1.035]	0.7631	0.2472	0.0488
SB	0.556 [0.509; 0.643]	0.556 [0.506; 0.643]	0.8775	0.1174	0.0249
MDA	6.88 [5.98; 8.72]	7.01 [5.90; 8.97]	0.7681	0.2077	0.1068
GGT	21.5 [18.3; 24.2]	21.3 [18.7; 24.2]	0.7838	0.1435	0.0029
SM	0.103 [0.072; 0.169]	0.100 [0.070; 0.141]	0.3915	0.1508	0.2261
SOD	60.5 [34.2; 81.6]	60.5 [31.6; 110.5]	0.5934	0.1973	0.2071
α-Amylase	288.4 [75.0; 398.0]	235.0 [117.0; 423.8]	0.5515	0.1406	0.1888
Lactate	2.41 [1.81; 3.44]	2.06 [1.45; 2.96]	0.0371	0.0447	0.7231
AOA	1.83 [1.43; 2.04]	1.63 [1.39; 1.99]	0.1716	0.0708	0.3922
Peroxidase	0.470 [0.315; 0.845]	0.400 [0.190; 0.710]	0.1366	0.9034	0.0497
MM280/254	0.871 [0.788; 1.025]	0.866 [0.774; 0.949]	0.2608	0.1451	0.4510
