# Supplementary figures and images for: Salivary Metabolites in Breast Cancer and Fibroadenomas: Focus on Menopausal Status and BMI
Source: Metabolites. 2024 Sep 30;14(10):531. doi: 10.3390/metabo14100531 (PMC11509358; doi:10.3390/metabo14100531)

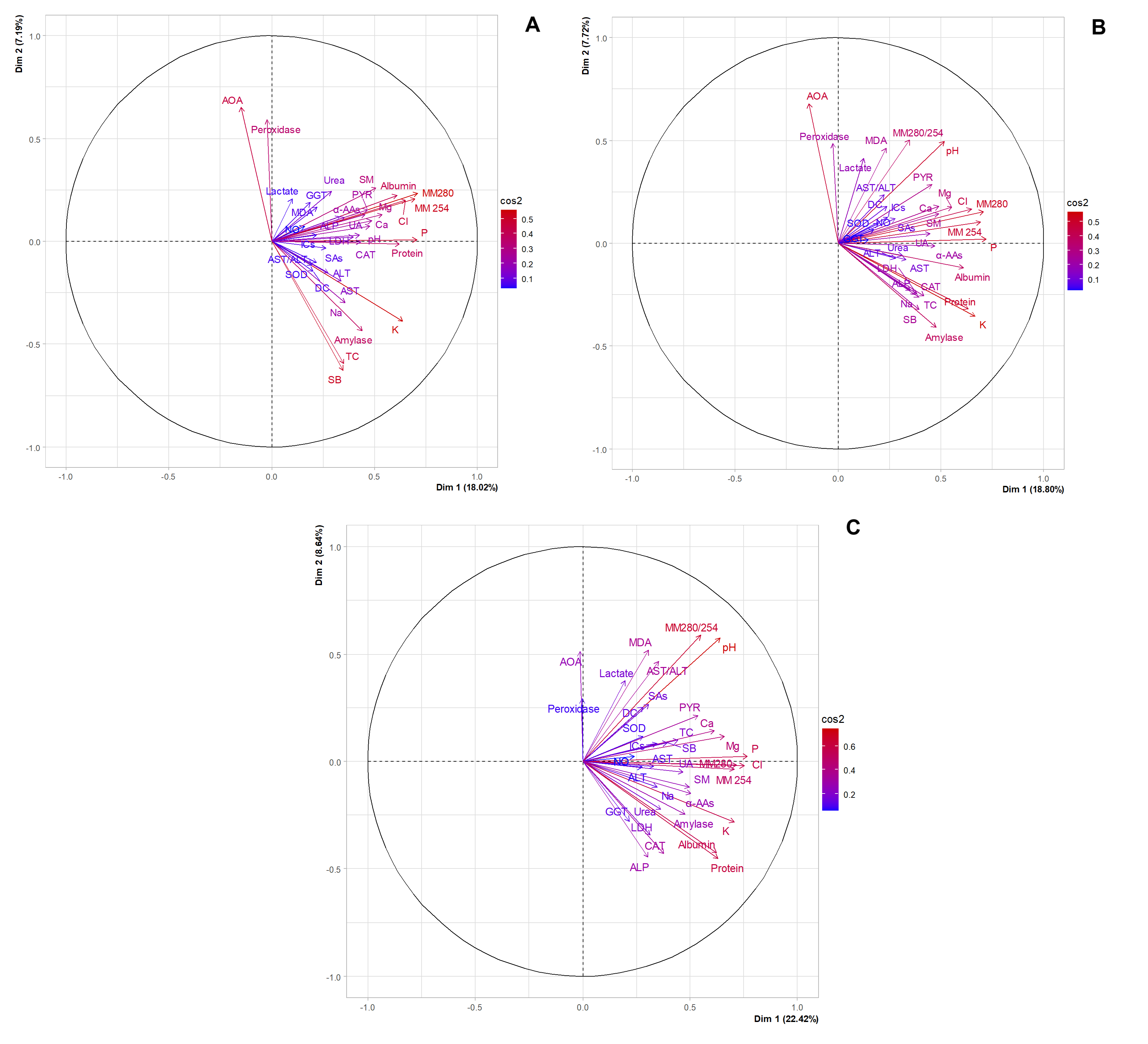

Supplement: Supplementary file 1 [file metabolites-14-00531-s001.zip › Bel'skaya_Figure_1S.tif]
